# Supplementary material for: Rapid evolution and host immunity drive the rise and fall of carbapenem resistance during an acute Pseudomonas aeruginosa infection
Source: Nat Commun. 2021 Apr 28;12:2460. doi: 10.1038/s41467-021-22814-9 (PMC8080559; doi:10.1038/s41467-021-22814-9)
Supplement: Supplementary file 1 — Supplementary Information [file 41467_2021_22814_MOESM1_ESM.pdf]

Supplementary Information

Primer name	Sequence 5'→3'	Use	Reference
rpsl-1	GCTGCAAAACTGCCCGCAACG	mRNA *rpsL* qRT-PCR	Oh H *et al.*, 2003 (1)
rpsl-2	ACCCGAGGTGTCCAGCGAACC		
mexB-U	CAAGGGCGTCGGTGACTTCCAG	mRNA *ampC*qRT-PCR	Juan C *et al.*, 2006 (2)
mexB-L	ACCTGGGAACCGTCGGGATTGA		
acrna-F	GGGCTGGCCTCGAAAGAGGAC	mRNA *mexB* qRT-PCR	Oh H *et al.*, 2003 (1)
acrna-R	GCACCGAGTCGGGGAACTGCA		
plas-1-F	GTATTTCTGCGCGACGTTCC	p110820 transformation screening	This study
plas-1-R	CTTCGATGAGTACCGGGTCG		
plas-2-F	CTCGGTCATGCTACGGAGAC		
plas-2-R	GAGTATGCGACCGATGAGCA		
plas-3-F	GTGCGATGGTTGATCGCTTC		
plas-3-R	GCCGTGGACGAATATCCCAT		

Supplementary Table 1. Primers used in study. Refere

nces for Supplementary Information

1. Oh, H., S. Stenhoff, S. Jalal, and B. Wretlind. Role of efflux pumps and mutations in genes for topoisomerases II and IV in fluoroquinolone-resistant *Pseudomonas aeruginosa* strains. *Microb. Drug Resist.* 8:323–328 (2003).
2. Juan, C., B. Moyà, J. L. Pérez, and A. Oliver. Stepwise upregulation of the *Pseudomonas aeruginosa* chromosomal cephalosporinase conferring high-level beta-lactam resistance involves three AmpD homologues. *Antimicrob. Agents Chemother.* 50:1780–1787 (2006).
