# Supplementary material for: Rapid evolution and host immunity drive the rise and fall of carbapenem resistance during an acute Pseudomonas aeruginosa infection
Source: Nat Commun. 2021 Apr 28;12:2460. doi: 10.1038/s41467-021-22814-9 (PMC8080559; doi:10.1038/s41467-021-22814-9)
Supplement: Supplementary file 3 — Reporting Summary [file 41467_2021_22814_MOESM3_ESM.pdf]

nature research

Corresponding author(s): R. Craig MacLean

Last updated by author(s): 21/3/2021

Reporting Summary

Nature Research wishes to improve the reproducibility of the work that we publish. This form provides structure for consistency and transparency in reporting. For further information on Nature Research policies, see our Editorial Policies and the Editorial Policy Checklist.

Statistics

For all statistical analyses, confirm that the following items are present in the figure legend, table legend, main text, or Methods section.

n/a	Confirmed	
☐	☒	The exact sample size (n) for each experimental group/condition, given as a discrete number and unit of measurement
☐	☒	A statement on whether measurements were taken from distinct samples or whether the same sample was measured repeatedly
☐	☒	The statistical test(s) used AND whether they are one- or two-sided<br>*Only common tests should be described solely by name; describe more complex techniques in the Methods section.*
☒	☐	A description of all covariates tested
☒	☐	A description of any assumptions or corrections, such as tests of normality and adjustment for multiple comparisons
☐	☒	A full description of the statistical parameters including central tendency (e.g. means) or other basic estimates (e.g. regression coefficient) AND variation (e.g. standard deviation) or associated estimates of uncertainty (e.g. confidence intervals)
☐	☒	For null hypothesis testing, the test statistic (e.g. F, t, r) with confidence intervals, effect sizes, degrees of freedom and P value noted<br>*Give P values as exact values whenever suitable.*
☒	☐	For Bayesian analysis, information on the choice of priors and Markov chain Monte Carlo settings
☒	☐	For hierarchical and complex designs, identification of the appropriate level for tests and full reporting of outcomes
☒	☐	Estimates of effect sizes (e.g. Cohen's d, Pearson's r), indicating how they were calculated

Our web collection on statistics for biologists contains articles on many of the points above.

Software and code

Policy information about availability of computer code

Data collection	Data was entered in a web-based electronic data capture system that was designed for ASPIRE-ICU. The study site entered data in the system from the subject's source documents (i.e. medical chart).
Data analysis	JMP v.12 was used for statistical analysis. The following programs were used for sequence analysis: canu v. 1,8, circlator v.1.5.5 , trimmomatic v. 0.39, SPAdes v. 3.13.1, pilon v. 1.23, prokka v. 1.14.0, Bowtie 2 v2.2.4, SAMtools v0.1.16, PicardTools v1.140, Genome Analysis Toolkit (GATK) v3.4.46, SnpEff v4.2, bwa v. 0.7.17, BCFtools v. 1.9, breseq v. 0.34.0, GenAPI v. 1.0

For manuscripts utilizing custom algorithms or software that are central to the research but not yet described in published literature, software must be made available to editors and reviewers. We strongly encourage code deposition in a community repository (e.g. GitHub). See the Nature Research guidelines for submitting code & software for further information.

Data

Policy information about availability of data

All manuscripts must include a data availability statement. This statement should provide the following information, where applicable:

- Accession codes, unique identifiers, or web links for publicly available datasets
- A list of figures that have associated raw data
- A description of any restrictions on data availability

-All sequence data for the manuscript has been uploaded to NCBI's short-read archive (PRJNA667268)
-All data have been uploaded to Nature's Research Data
-All clinical data from the patient that were analyzed as part of the ASPIRE-ICU study have been included in this manuscript.

Field-specific reporting

Please select the one below that is the best fit for your research. If you are not sure, read the appropriate sections before making your selection.

- [ ] Life sciences
- [ ] Behavioural & social sciences
- [x] Ecological, evolutionary & environmental sciences

For a reference copy of the document with all sections, see nature.com/documents/nr-reporting-summary-flat.pdf

Ecological, evolutionary & environmental sciences study design

All studies must disclose on these points even when the disclosure is negative.

Study description

This study investigated the evolutionary responses to antibiotic treatment in a single patient using novel 'high definition' approaches.

Research sample

The study uses data and samples from a single patient. Samples were collected from the patient (peri-anal swabs or entotracheal aspirate) and then processed to obtain individual bacterial isolates (n=12 per sample).

Sampling strategy

This patient was enrolled in the ASPIRE-ICU study, and the same sampling strategy was used for all patients enrolled in the trial. Detailed information on this trial is cited in the text. Briefly, samples (endotracheal aspirate and peri-anal swabs) were collected at regularly defined intervals for all patients enrolled in the trial, and supplementary samples were collected from all patients who developed pneumonia during the trial (including this patient). The number of bacterial isolates collected from each clinical sample (12 randomly chosen isolates, or all isolates from samples with <12 colonies) was the same for all samples in this study.

Data collection

Clinical data for the patient was recorded by staff at the Virgen Macarena Hospital (Sevilla, Spain). Data on antibiotic use during the ICU stay and in the preceding two weeks was recorded. Respiratory samples and and peri-anal swabs were collected on the following visit days: 1 (the day of informed consent and study enrollment, 72h after ICU admission), 4, 7, and twice weekly for 30 days or until ICU discharge. In this case: day 10, 13, 16, 21, 23, 27. From patients who were diagnosed with pneumonia, additional respiratory samples were collected at the day of diagnosis and 7 days post-infection: day 2 and 8.
Peri-anal swabs in skimmed milk medium and untreated respiratory samples were stored at -80 °C until shipment to the Central lab at the University of Antwerp and until further analysis. Semi-quantitative culture of peri-anal swabs was performed by inoculating the swabs directly on CHROMID P. aeruginosa Agar (BioMérieux, France) and blood agar (BBL®Columbia II Agar Base (BD Diagnostics, USA) supplemented with 5% defibrinated horse blood (TCS Bioscience, UK)). After incubation of 24 h at 37 °C, the growth of P. aeruginosa was evaluated in four quadrants. Plates without growth were further incubated for 48 h and 72 h.

Patient ETA samples were blended (30,000 rpm, probe size 8 mm, steps of 10 s, max 60 s in total), diluted 1:1 v/v with Lysomucil (10% Acetylcysteine solution) (Zambon S.A, Belgium) and incubated for 30 minutes at 37 °C with 10 s vortexing every 15 minutes. Thereafter, quantitative culture was performed by inoculating 10-fold dilutions on CHROMID P. aeruginosa Agar and blood agar using spiral plater EddyJet (IUL, Spain). Plates were incubated at 37 °C for 24 h and CFU/mL was calculated. Plates without growth were further incubated for 48 h and 72 h. Matrix-Assisted Laser Desorption Ionization-Time of Flight Mass Spectrometry (MALDI-TOF MS) was used to identify 12 colonies per sample which were stored at -80 °C until further use.

Timing and spatial scale

Samples were collected at days 1,2,4,7,8,10,13,16,21,23 and 27 following enrollment in the trial. Actual dates will not be provided as these could compromise patient anonymity in conjunction with data reported in the manuscript.

Data exclusions

One isolate was excluded from analysis because whole-genome sequencing revealed that this isolate was from a different species (Staphylococcus epidermidis)

Reproducibility

All experiments reported in this manuscript were repeated, as outline in the methods.

Randomization

MIC assays: We assayed the antibiotic susceptibility of respiratory and peri-anal isolates as separate groups. We measured the resistance of each isolated to each antibiotic in 3 independent assays.
Colistin tolerance assays: We analysed the colistin tolerance of 55 respiratory isolates using a randomized block experimental design. Each isolate was randomly assigned to 1 of 5 blocks, and each block included 5 replicates of a control lab strain.

Growth rate assays: We measured the growth rate of all 60 respiratory isolates in 10 replicate assays.

Blinding

All bacterial isolates were assigned a unique identifier that did not reflect the sample type (ie ETA or peri-anal), or time point.

Did the study involve field work? [ ] Yes [x] No

Reporting for specific materials, systems and methods

We require information from authors about some types of materials, experimental systems and methods used in many studies. Here, indicate whether each material, system or method listed is relevant to your study. If you are not sure if a list item applies to your research, read the appropriate section before selecting a response.

Materials & experimental systems

n/a	Involved in the study
☒	☐ Antibodies
☒	☐ Eukaryotic cell lines
☒	☐ Palaeontology and archaeology
☐	☒ Animals and other organisms
☐	☒ Human research participants
☐	☒ Clinical data
☒	☐ Dual use research of concern

Methods

n/a	Involved in the study
☒	☐ ChIP-seq
☒	☐ Flow cytometry
☒	☐ MRI-based neuroimaging

Animals and other organisms

Policy information about studies involving animals; ARRIVE guidelines recommended for reporting animal research

Laboratory animals

For laboratory animals, report species, strain, sex and age OR state that the study did not involve laboratory animals.

Wild animals

Provide details on animals observed in or captured in the field; report species, sex and age where possible. Describe how animals were caught and transported and what happened to captive animals after the study (if killed, explain why and describe method; if released, say where and when) OR state that the study did not involve wild animals.

Field-collected samples

For laboratory work with field-collected samples, describe all relevant parameters such as housing, maintenance, temperature, photoperiod and end-of-experiment protocol OR state that the study did not involve samples collected from the field.

Ethics oversight

Identify the organization(s) that approved or provided guidance on the study protocol, OR state that no ethical approval or guidance was required and explain why not.

Note that full information on the approval of the study protocol must also be provided in the manuscript.

Human research participants

Policy information about studies involving human research participants

Population characteristics

Subjects eligible to be enrolled in the study were adult patients admitted to the ICU with an anticipated length of stay of at least 48 hours, who were undergoing mechanical ventilation at ICU admission (or expected to be within 24 hours thereafter). In eligible subjects, S. aureus colonization was ascertained in the nose and lower respiratory tract at ICU admission. S. aureus–colonized and non-colonized patients were enrolled into the study cohort in a 1:1 ratio. Additionally, for a patient to be included it was necessary that within 72 hours after the start of the first episode of mechanical ventilation both the SA colonization status was known and the written informed consent was obtained .

All the relevant characteristics of the included participant were reported in the manuscript

Recruitment

The following exclusion criteria were applied for the study cohort:
• Previous participation as a subject in the study cohort;
• Simultaneous participation of the subject in any preventive experimental study into anti-staphylococcus or anti-pseudomonas interventions; and
• Expected death (moribund status) within 48h, or ICU discharge of the participant within 24h, at the moment of informed consent.
We do not consider that these exclusion criteria had any bias on the results of this study.

Ethics oversight

The ASPIRE-ICU study was approved by the Andalusian Biomedical Research Ethics Coordinating Committe (CCEIBA).

Note that full information on the approval of the study protocol must also be provided in the manuscript.

Clinical data

Policy information about clinical studies
All manuscripts should comply with the ICMJEguidelines for publication of clinical research and a completedCONSORT checklist must be included with all submissions.

Clinical trial registration

ASPIRE-ICU ClinicalTrials.gov Identifier: NCT02413242

Study protocol

Paling, F.P., et al., Rationale and design of ASPIRE-ICU: a prospective cohort study on the incidence and predictors of Staphylococcus aureus and Pseudomonas aeruginosa pneumonia in the ICU. BMC infectious diseases, 2017. 17(1): p. 643 (the full ASPIRE-ICU protocol can be obtained upon request).

Data collection

ASPIRE-ICU was a cohort study of adult ICU patients at 30 hospitals in 11 European countries that recruited participants between June 2015 and October 2018.

Outcomes

The primary outcome (Pseudomonas aeruginosa pneumonia acquired in the ICU) was assessed in multiple steps. First, the following 4

clinical criteria were assessed daily: any new antibiotic use, new blood cultures performed, new chest radiograph or computed tomography scan that shows a new or worsening infiltrate, or other new reason to suspect pneumonia. In cases of at positive answer, a combination of objective major and minor criteria was assessed to categorize patients as having protocol-defined pneumonia or not, as described elsewhere. The primary end point was determined post hoc on the basis of isolation of P. aeruginosa from any lower respiratory tract specimen (including both clinical and study surveillance cultures) or blood culture in the 3 days before and after the day of pneumonia diagnosis, according to clinical criteria. Death was assessed at ICU discharge, at day 30 and day 90 after ICU admission. No other secondary outcomes were evaluated.
